# Supplementary material for: The p48 isoform of the PA2G4/EBP1/ITAF45 oncoprotein is required for the encephalomyocarditis virus IRES-driven translation initiation
Source: Nucleic Acids Res. 2025 Dec 3;53(22):gkaf1281. doi: 10.1093/nar/gkaf1281 (PMC12673855; doi:10.1093/nar/gkaf1281)
Supplement: gkaf1281_Supplemental_Files [file gkaf1281_supplemental_files.zip › Kushchenko_ITAF45_Supplementary_Data.pdf]

The p48 isoform of the PA2G4/EBP1/ITAF45 oncoprotein is required for the encephalomyocarditis virus IRES-driven translation initiation

Artem S. Kushchenko, Violetta A. Golovko, Eugenia A. Panova, Anastasia P. Sukhunina, Ekaterina E. Gladneva, Alexandr Y. Krasota, Yury Y. Ivin, Anastasia V. Poteryakhina, Vadim I. Agol, Sergey E. Dmitriev

SUPPLEMENTARY DATA

Supplementary Table 1. Top six sgRNA hits from a genome-wide knockout CRISPR screen in HEK293T cells identifying host genes involved in EMCV/Mengo virus infection. Read counts from the Illumina sequencing library are shown. *PA2G4* and *ADAM9*, as mentioned in the main text, are highlighted.

Gene	Count	Sequence
PA2G4	903	CTCCCCTTTGAAGAGCGACC
ADAM9	681	TTAGGTATCTTATGTTATTC
CSE1L	622	CGTTTGACTTAAATTCATGA
PAX7	320	GCCGGATGGACCCGGTCTCC
RREB1	239	GACAATCGCCTACGTTTCAG
CT47A9	93	GCTGGTGTCATGTCTGCCAC

```
WT        CTACATTTCTGAAGGGCACTAGGGCTCCCGGGAGACAGCAAGGCAGTAGGCTGATGATTCTTTCTTTACAGGTATTGCTTTTCCCACCAGCATTTCGGTAAATAACTGTGTATGTCACTTCTCCCCTTTGAAGAGCGACCAGGATTA
allele #1 CTACATTTCTGAAGGGCACTAGGGCTCCCGGGAGACAGCAAGGCAGTAGGCTGATGATTCTTTCTTTACAGGTATTGCTTTTCCCACCAGCATTTCGGTAAATAACTGTGTATGTCACTTCTCCCCT--------CGACCAGGATTA (Δ 8 bp)
allele #2 CTACAT-----------------------------------------------------------------------------------------------------------------------------------ACCAGGATTA (Δ 131 bp)
```

Supplementary Figure 1. Nucleotide sequences of the *ITAF45* gene locus in WT HEK293T cells and in the *ITAF45* KO cell line (alleles 1 and 2 shown). The second intron sequence is shown in blue, and the third exon in green. Nucleotides corresponding to the sgRNA used for CRISPR-mediated genome editing are underlined. In allele-1, an 8-bp deletion occurred, shifting the reading frame at the beginning of the third exon. Allele-2 has a 131-bp deletion disrupting the intron-exon junction and affecting pre-mRNA splicing.

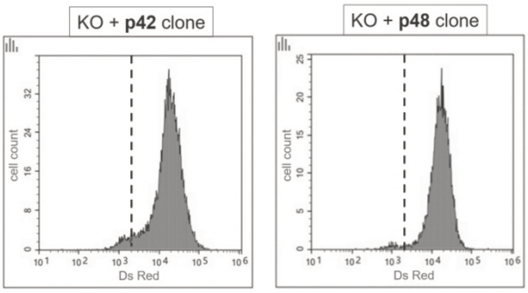

Supplementary Figure 2. Validation of ITAF45-KO+p42 and ITAF45-KO+p48 monoclonal cell lines for homogeneous transgene expression across the cell population. The mCherry gene present in the lentiviral cassette was used as a marker of transgene expression. Representative flow cytometry plots for the two cell lines are shown. mCherry-negative cells are to the left of the dashed line, whereas the vast majority of cells are mCherry-positive.

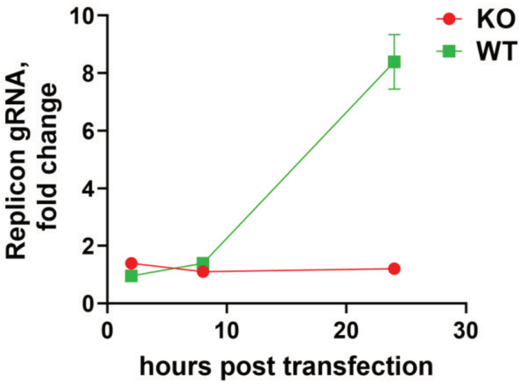

Supplementary Figure 3. Mengo replicon RNA accumulation in WT and KO cells transfected with in vitro-transcribed replicon RNA, measured at 2, 8, and 24 hpt by RT-qPCR. The experiments were conducted in three biological replicates, with qPCR reactions performed in four technical replicates. The means ± SD are shown.

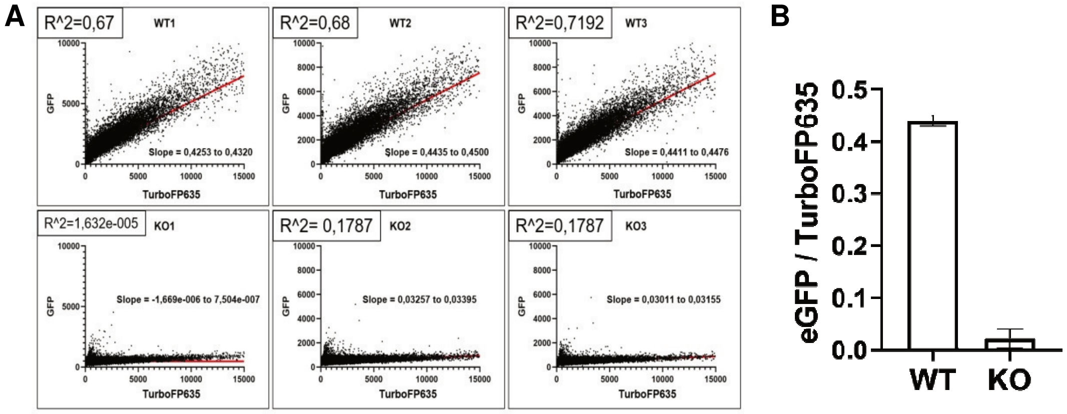

Supplementary Figure 4. Flow cytometry data for WT and ITAF45-KO HEK293T cells transfected with capped and polyadenylated KAT-EMCV_IRES-EGFP mRNA. (A) Flow cytometry plots from three biological replicates are shown. The analysis was performed 24 h after transfection. A linear regression was performed on the eGFP/TurboFP635 signal. R^2 and slope coefficients are shown. (B) Slope coefficients from three independent experiments (mean ± SD) are shown.
